# Longitudinal Strain vs. Conventional Echocardiographic Parameters in the First Week of Life in Healthy Term Newborns

**DOI:** 10.1007/s00246-023-03291-x

**Published:** 2023-12-08

**Authors:** Jerneja Rešek Peček, Mirta Koželj, Petja Fister

**Affiliations:** 1 Department of Pediatric Critical Care, Children’s Hospital, University Medical Centre Ljubljana, Bohoričeva 20, 1000 Ljubljana, Slovenia; 2 Unit of Cardiology, Children’s Hospital, University Medical Centre Ljubljana, Ljubljana, Slovenia; 3 Faculty of Medicine, University of Ljubljana, Ljubljana, Slovenia

**Keywords:** Echocardiography, Longitudinal strain, Newborn, Speckle tracking

## Abstract

The first week of life is characterized by substantial alterations in hemodynamic conditions. Changes in myocardial contractility will reflect these changes. We aimed to assess right and left ventricular function on the third and seventh days of life in 50 healthy term newborns. To assess myocardial function, we used speckle tracking echocardiography. Pulsed-wave tissue Doppler imaging, M-mode, Doppler and pulsed-wave Doppler were also used to assess ventricular function. We found a significant increase in both right and left longitudinal strain and an increase in systolic and diastolic tissue Doppler velocities, whereas most other parameters remained unchanged. At both time points, the measured parameters were significantly greater for the right ventricle, but the changes with time were similar for both ventricles. We also found an increase in right ventricular outflow tract acceleration time as an indirect sign of decreasing pulmonary vascular resistance and an increase in systolic blood pressure, pointing to increasing systemic vascular resistance. Together with a decreasing proportion of patients with patent ductus arteriosus, the estimated left ventricular cardiac output decreased and right ventricular cardiac output increased but not to a statistically significant degree. In conclusion, the results of our study show how different echocardiographic techniques capture hemodynamic changes and changes in myocardial contractility and compliance. Both longitudinal strain and tissue Doppler imaging parameters seem to offer greater sensitivity in comparison with conventional echocardiographic parameters.

## Introduction

After birth, pulmonary and systemic circulation separate and run serially, as opposed to two parallel circulations in utero, and the role of the dominant ventricle must be taken over by the left ventricle (LV) instead of the right ventricle (RV). At the same time, many changes in cardiac metabolism, structure and electrochemical properties as well as hormonal milieu are taking place, not just in the first hours after birth but over several days and weeks of life, when the LV dominance pattern is slowly established [1–3].

As echocardiography plays an increasingly important role in daily clinical practice in the assessment of cardiac function and management of neonates, it is important to examine how physiological hemodynamic and contractile changes affect quantitative echocardiographic measurements of RV and LV function. While the development of cardiac function during infancy and childhood is well characterized, attention has only recently focused on the changes that occur in the early postnatal period [4–6]. Prospective studies of changes in myocardial function, detected by echocardiography in the first weeks of life, are rare and include small sample sizes [7]. Scholars in previous studies chose a longer longitudinal follow-up, where some increases may be growth-dependent [8], or studied longitudinal changes in myocardial function in preterm newborns [9].

In our study, we wanted to assess how RV and LV function change between the third and seventh days of life in healthy term newborns and how these changes in functional parameters differ between the ventricles. Our primary goal was to analyze RV and LV longitudinal strain parameters as novel quantitative measures of ventricular function that demonstrated high clinical feasibility and reproducibility in previous studies [10–12]. The secondary objective of our study was to evaluate some of the more conventional echocardiographic parameters derived from pulsed-wave tissue Doppler imaging (PW-TDI), M-mode and pulsed-wave Doppler analysis of ventricular filling.

With our study, we hope to provide an understanding of how different echocardiographic techniques capture the aforementioned changes in cardiac physiology after birth and gain a better understanding of RV and LV function in newborns.

## Patients and Methods

A prospective observational study on a group of 50 newborns was conducted between November 2018 and July 2022 at the Department of Pediatric Critical Care and the Department of Neonatology, Children’s Hospital, University Medical Center Ljubljana, Slovenia. The parents gave their informed consent, and ethical approval was obtained from the National Medical Ethics Committee (0120-671/2017/9). The study was conducted in accordance with the principles outlined in the Declaration of Helsinki.

Each newborn underwent two serial evaluations by echocardiography: the first measurement was performed on the third day of life, and the second measurement was performed on the seventh day of life. A simultaneous electrocardiogram was also recorded, and the blood pressure was measured noninvasively using an inflatable cuff and electronic sphygmomanometer.

Newborns were selected based on the following inclusion criteria: gestational age 37–42 weeks, singleton birth, uncomplicated pregnancy and delivery and appropriate birth weight for gestational age (10th to 90th percentiles). We excluded newborns with clinical signs or personal history suggestive of perinatal distress, signs of infection or cardiorespiratory pathology and incidental findings of congenital heart defects on echocardiography, except for hemodynamically insignificant patent ductus arteriosus (PDA) or patent foramen ovale (PFO).

### Quantitative Echocardiographic Evaluation

All echocardiograms were performed by a single investigator and according to the practice guidelines and recommendations of the Writing Group of the American Society of Echocardiography in collaboration with the European Association of Echocardiography and the Association for European Pediatric Cardiologists [13]. A Vivid S70 device was used (GE Healthcare, Horten, Norway) with a 4.0–12.0 MHz phased-array transducer.

Echocardiograms were acquired during rest or sleeping to minimize crying and without using any sedation of the newborn.

All echocardiograms were digitally stored, and subsequent quantitative analyses were performed by one investigator, following the recommendations for quantification of the American Society of Echocardiography [14], using EchoPAC Clinical Workstation Software version 203, GE Healthcare.

RV and LV systolic function was quantitatively assessed by measuring peak RV free wall and global longitudinal strain (RV FWLS and RV GLS) and LV global longitudinal strain (GLS) by two-dimensional speckle tracking echocardiography (2D STE) from the RV- and LV-focused apical four chamber view [10]. The frame rate of image acquisition was 96 frames per second for a frame rate-to-heart rate ratio between 0.7 and 0.9 frames per second per beat per minute. We chose to include the septum in the measurement of LV function as part of the GLS because although it contributes to the function of both ventricles, it is currently regarded as part of the LV [15, 16].

We also analyzed the following conventional echocardiographic parameters: tricuspid annular plane systolic excursion (TAPSE), mitral annular plane systolic excursion (MAPSE), peak systolic (*s*ʹ) PW-TDI myocardial velocity at the lateral tricuspid valve annulus (tricuspid annular plane systolic velocity) and at the lateral mitral valve annulus (mitral annular plane systolic velocity), peak early (*e*ʹ) and late (*a*ʹ) diastolic PW-TDI velocities at the tricuspid and mitral annulus and pulsed-wave Doppler early (*E*) and late (*A*) inflow velocities of the tricuspid valve (TV) and mitral valve (MV). We calculated the *E*/*A*, *e*ʹ/*a*ʹ and *E*/*e*ʹ ratios. Global RV and LV function were evaluated using the PW-TDI-derived myocardial performance index (MPIʹ), and cardiac output was estimated by measuring the RV and LV outflow tract velocity time integral (RVOT-VTI and LVOT-VTI).

For the estimation of RV afterload (pulmonary arterial pressure), RV outflow tract acceleration time (RVOT-AT) was measured by pulsed wave Doppler at the level of the pulmonary valve and adjusted for heart rate by dividing it by the square root of the RR interval [17].

Each parameter was measured on three consecutive cardiac cycles and subsequently averaged. For any given parameter, measurements were made only if satisfactory and unambiguous views were available. Technically inadequate recordings with poor image quality were excluded from the analysis.

### Statistical Methods

Approximately normally distributed continuous variables are presented as the means and standard deviations and as medians and interquartile ranges otherwise. Categorical variables are presented as frequencies and percentages. The difference in clinical and hemodynamic parameters of two consecutive measurements was tested by paired sample *t* test or Wilcoxon test for continuous variables and by McNemar test for categorical variables. Mixed-way ANOVA was used to test the time, ventricle and interaction effects for all functional parameters. Pearson’s correlation coefficient was used to test possible correlations between parameters. The comparisons with *p* < 0.05 were treated as statistically significant. All analyses were performed using SPSS, version 28.

## Results

The characteristics of the study population are presented in Table 1, and clinical and hemodynamic parameters on the third and seventh days of life are shown in Table 2.

**Table 1:** Characteristics of the study population

	Study population (*n* = 50)
Male, *n* (%)	31 (60)
Gestational age, weeks, mean ± SD,	39 ± 1.6
Birth weight, grams, mean ± SD	3406 ± 561
Birth by cesarean section, *n* (%)	13 (26)
Apgar score at 1 min, median (IQR)	9 (0)
Apgar score at 5 min, median (IQR)	9.5 (1)

*n* number, *SD* standard deviation, *IQR* interquartile range

**Table 2:** Clinical and hemodynamic parameters of two consecutive measurements on the third (1) and the seventh (2) day of life

	1	2	*p* value
Weight, grams, mean ± SD (*n* = 49)	3296 ± 528	3439 ± 586	<0.001
PDA, *n* (%)	13 (26)	3 (6)	0.002
Systolic blood pressure, mmHg, mean ± SD (*n* = 33)	72 ± 7	76 ± 7	0.012
Diastolic blood pressure, mmHg, mean ± SD (*n* = 33)	43 ± 7	44 ± 7	0.547
Mean blood pressure, mmHg, mean ± SD (*n* = 34)	51 ± 11	53 ± 11	0.180
Heart rate/min, mean ± SD (*n* = 50)	124 ± 19	133 ± 17	0.009

*SD* standard deviation, *PDA* patent ductus arteriosus, *n* number

The proportion of patients with PDA decreased with time. Systolic blood pressure and heart rate at the time of the echocardiographic examination were significantly higher on the 7th day of life.

Table 3 summarizes functional parameters on the third and seventh days of life for the right and left ventricles while also presenting the number of newborns included in the analysis and the results of mixed-way ANOVA.

**Table 3:** Functional parameters of the right and left ventricles evaluated by echocardiography on the third (1) and the seventh (2) days of life and results of mixed-way ANOVA

	Right ventricle		*n*	Left ventricle		*n*	Time	Ventricle	Interaction
	1	2		1	2				
Systolic function									
RV FWS/LV GLS (%)	−19.28 ± 4.24	−20.58 ± 4	35	−14.1 ± 2.3	−14.9 ± 2	29	**0.005**	<0.001	0.503
GLS (%)	−16.47 ± 3	−17.51 ± 2.8	40	−14.1 ± 2.3	−14.9 ± 2	29	**0.001**	<0.001	0.672
TAPSE/MAPSE (cm)	0.92 ± 0.15	0.91 ± 0.18	41	0.57 ± 0.10	0.61 ± 0.11	40	0.371	<0.001	0.131
*s*ʹ (cm/s)	7.02 ± 1.2	7.7 ± 1.2	45	4.93 ± 1.2	5.53 ± 1.15	43	**<0.001**	<0.001	0.760
Diastolic function									
* E* (cm/s)	49.26 ± 10.87	48.03 ± 9.19	38	56.6 ± 10.97	57.31 ± 11.33	38	0.844	<0.001	0.468
* A* (cm/s)	60.79 ± 11.61	61.05 ± 12.19	38	53.39 ± 11.24	57.02 ± 11.53	38	0.150	0.016	0.213
* E*/*A*	0.83 ± 0.16	0.79 ± 0.17	38	1.1 ± 0.27	1.02 ± 0.16	38	**0.018**	<0.001	0.293
* e*ʹ (cm/s)	7.37 ± 1.31	7.99 ± 1.35	35	6.18 ± 1.77	6.69 ± 1.4	39	**0.003**	<0.001	0.774
* a*ʹ (cm/s)	9.11 ± 1.79	10.52 ± 2.12	34	6.31 ± 1.86	6.95 ± 1.58	43	**<0.001**	<0.001	0.09
* e*ʹ/*a*ʹ	0.83 ± 0.19	0.79 ± 0.18	33	1.03 ± 0.30	1.03 ± 0.29	39	0.563	<0.001	0.411
* E*/*e*ʹ	6.75 ± 1.83	6.17 ± 9.28	28	10.31 ± 5.07	9.28 ± 2.93	30	0.064	<0.001	0.602
Global function									
MPIʹ	0.40 ± 0.10	0.35 ± 0.10	36	0.52 ± 0.13	0.49 ± 0.11	37	**0.015**	<0.001	0.631
Cardiac output									
VTI (cm/s)	13.04 ± 2.81	14.17 ± 2.7	43	12.31 ± 1.87	11.52 ± 2.36	43	0.481	<0.001	**<0.001**
Afterload									
RVOT-AT adjusted to heart rate (ms)	113.17 ± 16.27	121.57 ± 20.32	38	–	–	–	**0.024**	–	–

Bold values indicate statistical significance at the *p* < 0.05 level

Data are presented as the mean ± SD

*RV FWLS* right ventricle free wall longitudinal strain, *LV GLS* left ventricle global longitudinal strain, *GLS* global longitudinal strain, *TAPSE* tricuspid annular plane systolic excursion, *MAPSE* mitral annular plane systolic excursion, *sʹ* peak systolic myocardial velocity at the lateral tricuspid/mitral valve annulus, *E* tricuspid/mitral valve early inflow velocity, *A* tricuspid/mitral valve late inflow velocity, *E/A* ratio of tricuspid/mitral valve early and late inflow velocity, *eʹ* peak early diastolic myocardial velocity at the lateral tricuspid/mitral valve annulus, *aʹ* peak late diastolic myocardial velocity at the lateral tricuspid/mitral valve annulus, *eʹ/aʹ* ratio of peak early and late diastolic myocardial velocity at the lateral tricuspid/mitral valve annulus, *E/eʹ* ratio of tricuspid/mitral valve early inflow velocity and peak early diastolic myocardial velocity at the lateral tricuspid/mitral valve annulus, *MPIʹ* right/left ventricle myocardial performance index, *VTI* right/left ventricle outflow tract velocity time integral, *RVOT-AT* right ventricular outflow tract acceleration time

The comparison of functional parameters between LV and RV showed statistically significant differences for all parameters. A statistically significant change between the third and the seventh day of life was present for both ventricles in longitudinal strain as measured by FWS/GLS (%) (*p* = 0.005) and GLS (*p* = 0.001), while the interaction effect of time and ventricle was not statistically significant (*p* = 0.503; *p* = 0.672, respectively). An insignificant interaction effect showed a lack of difference in the change of longitudinal strain from the third to the seventh day of life between the ventricles (both ventricles had similar changes in the functional parameters). Regarding systolic function, a statistically significant effect of time was also present for *s*ʹ (*p* < 0.001) and insignificant for TAPSE/MAPSE (*p* = 0.371). Regarding diastolic function, a statistically significant effect of time was found for the *E*/*A* ratio (*p* = 0.018), *e*ʹ (*p* = 0.003) and *a*ʹ (*p* < 0.001). There was a statistically significant change in RV/LV MPIʹ between the third and the seventh day of life for both ventricles (*p* = 0.015). The only statistically significant interaction effect between time and ventricle was found for RVOT/LVOT-VTI (*p* < 0.001). For the RV, the mean (SD) VTI increased from 13.04 (2.81) to 14.17 (2.7), and the VTI for the LV decreased from 12.31 (1.87) to 11.52 (2.36). Afterload of the RV as measured by RVOT-AT adjusted to heart rate significantly increased with time (*p* = 0.024).

Table 4 shows the correlation between RVOT-AT adjusted to heart rate and parameters of RV systolic function.

**Table 4:** Pearson’s correlation coefficient (*p* value; *n*) between functional parameters of the right ventricle and RVOT-AT adjusted to heart rate (the correlations are calculated for measurements made at the same time point)

	RVOT-AT	
	1	2
TAPSE	0.35 (0.052; 31)	−0.05 (0.793; 36)
TV *s*ʹ	0.27 (0.102; 39)	−0.12 (0.464; 39)
RV FWLS	0.03 (0.867; 29)	0.24 (0.21; 30)
RV GLS	0.04 (0.809; 35)	0.12 (0.493; 35)

*TAPSE* tricuspid annular plane systolic excursion, *TV sʹ* peak systolic myocardial velocity at the lateral tricuspid valve annulus, *RV FWLS* right ventricle free wall longitudinal strain, *RV GLS* right ventricle global longitudinal strain

No statistically significant correlation between RVOT-AT and other functional parameters on any of the measurements could be found.

## Discussion

Between the third and seventh day of life, we observed changes in RV and LV functional parameters that indicated an increase in systolic, diastolic and global function of both ventricles. At both time points, RV functional parameters were greater than LV parameters, but the increase in time was not different between the two ventricles.

RV and LV longitudinal strain parameters showed increases between the third and seventh days of life. Changes in both ventricles were also detected in PW-TDI-derived parameters. These observations could be at least partially the result of hemodynamic pressure changes in the observed period, at least for the RV, whose afterload decreased, evident from a statistically significant increase in RVOT-AT. However, we tested the correlation between RVOT-AT and parameters of RV systolic function and found no statistically significant positive correlation. Therefore, the adaptive maturation of the myocardium with changes in contractile properties and compliance must have had an effect on the observed variables. This also explains the increase in function of the LV, for which we can assume there was an increase in afterload, based on the known physiologic data about systemic vascular resistance and indicated by an increase in systolic blood pressure in our patients. Additionally, PW-TDI and strain-derived parameters could be influenced by heart rate, which was higher at the time of the second echocardiographic examination. We tried to minimize this impact by excluding recordings with fusion of PW-TDI diastolic velocity waves [18] and by optimizing image acquisition for longitudinal strain with the appropriate frame rate [19, 20].

No statistically significant differences in RV or LV function were captured by M-mode echocardiography or for most parameters measured by conventional pulsed-wave Doppler analysis of ventricular filling except for the *E*/*A* ratio. Likewise, neither RVOT nor LVOT-VTI changed significantly between the third and seventh days of life. This finding may point to a lower sensitivity of these conventional echocardiographic techniques and shows that newer methods, such as TDI and STE, may bring valuable information to the cardiac assessment in newborns by detecting changes in ventricular function earlier than conventional echocardiographic measures.

We found that tissue Doppler velocities (*s*ʹ, *e*ʹ and *a*ʹ waves) were higher for the RV as opposed to the LV (right vs. left lateral wall of the heart), which is similar to previous studies [18, 21–24]. It is also known that strain values are higher in the RV than in the LV, as we also found, reflective of the changing loading conditions specific to each ventricle [10, 25] and the different geometry of the ventricles [26], which also differ in anatomy, orientation of muscle fiber and muscle fiber histology. Specific RV contraction mechanics also impact the tissue Doppler measurements—the role of the RV inflow is more predominant in RV ejection than in the infundibular part [21]. *E*/*e*ʹ was higher on the left side than on the right side of the heart, which indicates a higher LV filling pressure and perhaps more immature diastolic properties of the left myocardium. However, *E*/*A* and *e*ʹ/*a*ʹ were >1 for the LV and <1 for the RV, which points to better diastolic function of the LV. The other possible reason for the higher *E*/*e*ʹ on the left side would be persistent ductus arteriosus [27], but it was hemodynamically significant in none of our patents.

When we analyzed the change in RV function vs. change in LV function, we found that they did not differ significantly; for almost all parameters, changes between the first and the second measurement were not significantly different between the ventricles. Thus, the function of both ventricles increased to a similar degree, even though the hemodynamic loading conditions of the ventricles changed in opposite directions. As already discussed, we could attribute this finding to intrinsic cardiomyocyte maturation after birth and hence improved contractile efficiency and increased compliance of the ventricles to enhance diastolic filling [1–3].

A notable exception was VTI, which we chose as a surrogate marker of cardiac output. LVOT-VTI decreased from the third to the seventh day of life, while RVOT-VTI increased, although not to a statistically significant degree. The trend of decreased LVOT-VTI could be due to an increase in afterload in a short period, when adaptation of the LV is not yet complete, and an increased afterload is thus followed by a decrease in cardiac output. Conversely, RV afterload decreased, and thus, RVOT-VTI increased. An inverse relationship between cardiac output and afterload has previously been shown in animal studies [28]. In explaining cardiac output changes, we must also consider the decrease in LV preload and increase in LV afterload that occurred with closure of the PDA with a decrease in left-to-right ductal shunting [29], whereas the flow through the PFO and associated right ventricular volume load generally remained unchanged throughout the observation period chosen in our study.

In our study, we chose RV and LV longitudinal strain parameters measured by STE as our primary focus. In recent years, there has been increasing interest in the application of strain measurements in the neonatal population since several studies indicate that strain is often a more sensitive and reproducible measure of ventricular function than conventional echocardiographic methods [6, 16, 30].

The relationship between age and strain values is an ongoing area of research, and normative data and reference values for strain in healthy neonates are still being established [31]. In general, it was reported that LV deformation parameters measured using 2D STE remain stable during the transitional period and up to 28 days. Khan et al. showed no significant difference between mean LV GLS in neonates from their meta-analysis and GLS in older subjects from previous meta-analyses [31], and Klitsie et al. did not find any significant changes in speckle tracking-derived LV strain parameters when prospectively studying healthy newborns 1–3 days, 3 weeks and 6–7 weeks after birth [31]. Similarly, no change in RV longitudinal strain was reported in the early transitional period by some studies [32, 33], but other authors found an increase in RV GLS and RV FWLS from 1 week to 1 month of age [20]. In a recently published study, Toma et al. did not observe variability in strain values with age, gestational age, sex, or weight [16]. In contrast, we previously reported an increase in RV longitudinal strain from the third to the seventh day of life in healthy term infants [6]. In this study, we found that this was also true for the LV. This is potentially due to the design of our study, where each newborn was assessed twice; thus, the variation between the individuals was eliminated, and the sensitivity of the statistical tests used was greater. In a study designed similarly to ours but involving use of TDI-derived strain and strain rates on a cohort of 105 preterm infants of less than 29 weeks gestation, James et al. were able to detect significant increases in RV strain values but not in LV or septum strain, comparing days 5–7 versus day 2 of life [9]. A smaller prospective study in 16 healthy term neonates by Kahr et al. also revealed significant increases in peak systolic strain in the LV and RV at three time points during the first month of life [7].

There are several limitations to our study. The first is the small sample size, tied to the capacities of our single center. We were forced to exclude some echocardiograms with poor image quality, which further decreased the number of newborns included in the analyses of certain parameters. We also chose to limit ourselves to a relatively narrow period, excluding major hemodynamic changes that occur in the first 72 h after birth. In future studies, potentially with a larger sample size, it would also be interesting to explore what happens between the seventh and 28th days of life.

## Conclusion

Within a short period of the first week of life, major changes occur in the function of both the right and left ventricles that are due to physiologic transition from the intrauterine environment, where hemodynamic conditions are completely different than postnatally, and accompany gradual adaptations in both blood flow and pressures that coincide with the adaptive maturation of the myocardium. The results of our study facilitate understanding how different echocardiographic techniques capture the changes in cardiac physiology after birth and provide a better basis for comprehending RV and LV function in newborns.

## Data Availability

The datasets used and/or analyzed during the current study are available from the corresponding author upon reasonable request.
